# Surface Electromyography for Evaluating the Effect of Aging on the Coordination of Swallowing Muscles

**DOI:** 10.1007/s00455-023-10572-3

**Published:** 2023-04-27

**Authors:** Wei-Han Chang, Mei-Hui Chen, Jen-Fang Liu, Wei Li Chung, Li-Ling Chiu, Yi-Fang Huang

**Affiliations:** 1 School of Traditional Chinese Medicine, Chang Gung University, Taoyuan City, 333323 Taiwan; 2 Department of Physical Medicine and Rehabilitation, Keelung Chang Gung Memorial Hospital, Keelung, 204201 Taiwan; 3 Department of Nutrition and Health Sciences, College of Human Ecology, Chang Gung University of Science and Technology, Taoyuan City, 333324 Taiwan; 4 Department of Physical Medicine and Rehabilitation, Linkou Chang Gung Memorial Hospital, Taoyuan City, 333423 Taiwan; 5 Department of Nutrition and Health Sciences and Research Center for Chinese Herbal Medicine, College of Human Ecology, Chang Gung University of Science and Technology, Taoyuan City, 333324 Taiwan; 6 Department of Nutrition and Health Sciences and Geriatric and Long-Term Care Research Center, College of Human Ecology, Chang Gung University of Science and Technology, No. 261, Wenhua 1st Rd, Guishan Dist, Taoyuan City, 333324 Taiwan; 7 Department of General Dentistry, Linkou Chang Gung Memorial Hospital, No. 5, Fuxing Street, Gueishan Dist, Taoyuan City, 333423 Taiwan; 8 School of Dentistry, College of Oral Medicine, Taipei Medical University, Taipei, 110301 Taiwan; 9 Graduate Institute of Dental and Craniofacial Science, College of Medicine, Chang Gung University, Taoyuan City, 333323 Taiwan

**Keywords:** Surface EMG, Swallowing, Dysphagia, Aging, Coordination, Pharyngeal phase

## Abstract

Swallowing function can deteriorate with age, leading to a risk of dysphagia. Swallowing evaluation by surface electromyography (sEMG) can be easily and extensively applied for an elderly population. This study evaluated the temporal events observed by sEMG to clarify how aging affects the coordination among the masticatory and suprahyoid muscles. We recruited elderly individuals (over 65 years old) who denied dysphagia. The sEMG activities of anterior temporalis, masseter, and suprahyoid muscles were recorded during 3, 15, and 30 ml water swallowing tests (WST). We calculated the time interval between anterior temporalis and suprahyoid peak activity (T-SH interval) and masseter and suprahyoid peak activity (M-SH interval) and analyzed their correlation with age. The subjects who could and could not swallow 30 ml of water in one gulp were further assigned into the one-gulp and piecemeal groups, respectively, for subgroup analysis. We recruited 101 subjects, among whom 75 (26 males and 49 females) were analyzed after excluding those with suspected dysphagia or low-quality sEMG recordings. Age was significantly correlated with the bilateral T-SH (left: *r* = 0.249, *p* = 0.031; right: *r* = 0.412, *p* < 0.01) and right M-SH (*r* = 0.242, *p* = 0.037) intervals in the 30 ml WST. The correlation between intervals and age were observed in both subgroups. sEMG can be used to investigate the effect of aging on the temporal coordination between masticatory and suprahyoid contraction. Further studies are needed to verify the validity of screening subclinical dysphagia in the elderly.

## Introduction

The effects of normal aging on swallowing function include a reduction in saliva production, tongue pressure, oral and pharyngeal sensitivity, swallowing muscle strength, upper esophageal sphincter opening, and pharyngeal elevation, impaired dental status, prolonged oral and pharyngeal phases, delayed triggering of the swallow reflex, and increased pharyngeal residuals and rate of penetration [1–3]. “Presbyphagia” is defined as age-related changes in swallowing physiology whose mechanisms can be accounted for by normal degeneration of the neuromuscular system [4]. Controversy surrounds presbyphagia’s role in dysphagia. Specifically, some researchers suggest that normal aging alone does not cause dysphagia [2, 4] while others infer it is a risk for dysphagia [1, 5]. Primary presbyphagia was thus proposed to define age-related changes of swallowing not leading to dysphagia, while secondary presbyphagia is linked to dysphagia [1, 6].

Numerous articles suggest that elders are susceptible to frailty and chronic disease, which deteriorate their swallowing condition. In elderly individuals with frailty, stroke, or Parkinsonism, some physiological characteristics of swallowing, such as prolonged bolus transit time, prolonged swallowing reflex latency [3, 7, 8], and delayed laryngeal closure [9], are correlated with aspiration pneumonia. In healthy elderly individuals, age-related swallowing changes, such as reduced tongue strength [10], geniohyoid muscle atrophy [11], and the position of the hyoid relative to the mandible [12], are also correlated with aspiration pneumonia. Elderly people with exaggerated age-related swallowing changes are prone to aspiration pneumonia.

On the other hand, a high proportion of healthy elderly individuals who denied dysphagia have abnormal findings in instrumental assessments [2, 13–15], suggesting that their mild dysphagia symptoms may be mistaken as normal aging and their risk of aspiration pneumonia is ignored. Therefore, the age-related swallowing changes of healthy community-dwelling elderly people must be systematically evaluated. To this aim, several portable and non-invasive instruments were investigated for dysphagia assessment, including tongue pressure meters, cervical auscultation, surface electromyography (sEMG) [16], and thyroid cartilage motion transducers [17, 18]. As swallowing is mediated by a neuromuscular system that can be assessed by sEMG [19], sEMG suitably evaluates the effect of aging on the motor control of swallowing. Specifically, sEMG yields magnitude and temporal parameters of muscle contraction, such as peak and mean amplitude, duration, the latency of time to peak, and the average median frequency [19, 20]. The recorded muscle activities could analyze the oral (orbicularis oris, masseter, and temporalis) and pharyngeal (submental and infrahyoid muscles) phases [19, 20]. Furthermore, these parameters could be used to screen for dysphagia and preliminarily differentiate its causes [19].

Temporal parameters of sEMG have been used to analyze the oral initial and swallowing reflex durations [21] and sequence of masticatory and submental muscle activities [22–24]. This indicates that sEMG can potentially evaluate how the central nervous system (CNS) controls the intricate sequences of swallowing. In contrast to temporal parameters, the amplitude parameters have been used to investigate the contractive intensity of each muscle group responding to swallowing different bolus viscosities [25] and the correlation of the intensity between different muscle groups [26].

Some articles have applied sEMG to verify the aging effect on masticatory [27–29] and swallowing movements (oral transit and pharyngeal phases) [21, 25, 30–32] in healthy elderly people. However, masticatory function is affected not only by the contractive force of the masticatory muscles but also by the dental condition and tongue function in the elderly [33], which limits the role of sEMG in assessing masticatory function. Regarding swallowing movement function, the range amplitude of the suprahyoid muscles, the duration of the infrahyoid muscles, the interval between the onset of suprahyoid and the offset of the infrahyoid muscles, and the starting time of the masseter and suprahyoid muscles (the interval between the onset of orbicularis oris activities and masseter/suprahyoid activities) were significantly longer in geriatric groups compared to younger groups [21, 25, 30–32]. However, those articles mostly addressed the effect of aging on the amplitude, duration, and starting time of a single muscle. Most suprahyoid muscles share the same CN V innervation with the masticatory muscles, except the geniohyoid muscle (which has CN XII innervation) [34]. In masticatory cycles, suprahyoid muscles work with masticatory muscles and anchor their origin in the hyoid bone to abduct the mandible. During swallowing, suprahyoid muscles with an anchored origin in the mandible contract to elevate the thyrohyoid complex, and masticatory muscles co-contract to stabilize the mandible [19, 22, 35]. To the best of our knowledge, no studies have examined the aging effect on the temporal dispersion of the co-contracture, which may reflect the coordination of the CNS control.

In the present study, we used sEMG to characterize the temporal parameters of the intervals between masticatory and suprahyoid muscles during different swallowing tasks. We hypothesized that normal aging affects the temporal coordination of the masticatory and suprahyoid muscles.

## Methods

### Subjects

All aspects of the study were approved by the Human Studies Research Committee, and written informed consent was obtained from each subject before recruitment. In the retrospective study, subjects were recruited from the elderly community and dental outpatient departments of a medical center from January of 2017 to January of 2018. Inclusion criteria were old adults (> 65 years old) who have (1) no dysphagia; (2) intact cognition; (3) intact communication abilities; and (4) a willingness to cooperate with the study procedures. Exclusion criteria were subjects who have (1) swallowing function disorders, including neurological disease, nasopharyngeal carcinoma, head and neck tumors, severe trauma history of the head and neck, and temporomandibular joint pain; (2) symptoms and signs of dysphagia in at least one of the swallowing tests; or (3) cannot complete the study procedure.

### EMG Recording

**Fig. 1:**
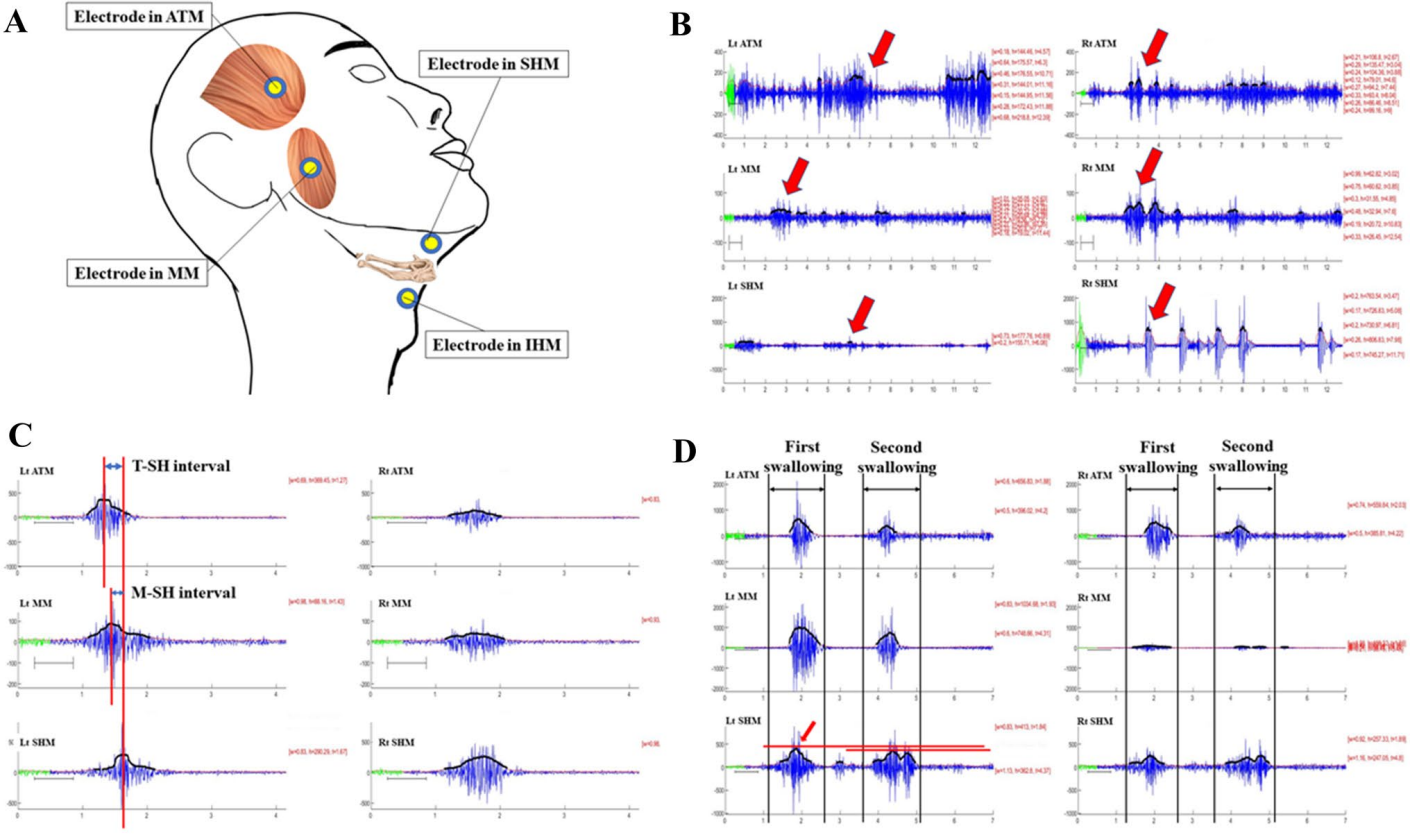
The location of electrode placement in the muscle belly of the anterior temporalis, masseter, and submental (suprahyoid muscles) and infrahyoid areas (infrahyoid muscles) (**A**). The low-quality trial without a series of activities of masticatory and suprahyoid muscles on the same side and symmetrical muscle activities in bilateral channels, compared with the high-quality trial (**B**, **C**). the peak-time intervals of the anterior temporalis and suprahyoid activities (T-SH interval), and the peak-time intervals of the masseter and suprahyoid activities (M-SH interval) measured by the timing interval of the maximal amplitude of masticatory and suprahyoid activities (**C**). The major series of activities of masticatory and suprahyoid muscles (first swallowing activities) with the highest maximal-amplitude in suprahyoid activities (**D**). (ATM: temporalis anterior muscle, MM: masseter muscle, SHM: suprahyoid muscles, IHM: infrahyoid muscles, T-SH: the peak-time intervals of the anterior temporalis and suprahyoid activities, M-SH: the peak-time intervals of the masseter and suprahyoid activities)

An eight-channel sEMG (K7x/EMG; Myotronics Inc., WA, USA) with silver-silver chloride, bipolar, and 1 cm interelectrode distance electrodes (Duotrode; Myotronics Inc., WA, USA) was used. Raw EMG signals were amplified, filtered, and computed with software (K7; Myotronics Inc., WA, USA). The signal envelope was calculated by the root-mean-square (RMS) with an envelope window of 200 ms. Electrodes were bilaterally attached to the skin along with the muscle belly of the anterior temporalis, masseter, and submental and infrahyoid areas (Fig. 1A) after the skin was thoroughly cleaned with alcohol pads. Laterality of swallowing movement in some normal population is the reason of bilateral recording in sEMG [36].

### Procedure

**Fig. 2:**
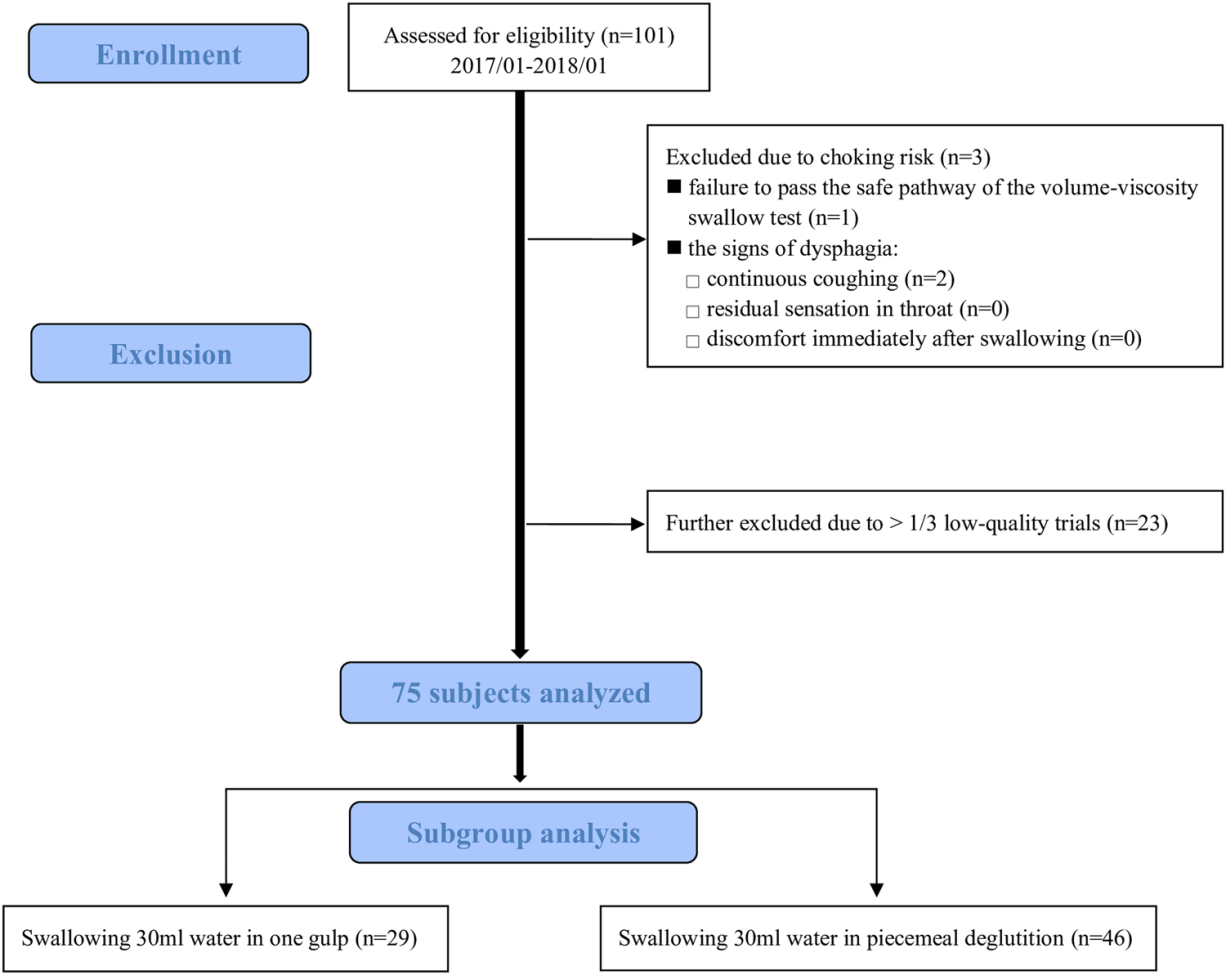
Flow diagram of participant enrollment, allocation, primary analysis, and subgroup analysis

The volume-viscosity swallow test (V-VST) [37] was first performed to screen the subjects’ dysphagia or aspiration. The subjects who completed the safe swallow pathway of V-VST would perform the water swallowing tests (WSTs) to evaluate the subject’s swallowing performance. Before WSTs, an EMG recording was applied and then each subject comfortably sat on a chair for five minutes to relax the swallowing muscles. If the subjects had signs of dysphagia, including continuous coughing, a residual sensation in the throat, or immediate post-swallowing discomfort during V-VST or WSTs, the procedure was stopped and the subjects were excluded (Fig. 2).

#### V-VST

The V-VST is a bedside method to screen patients for dysphagia [37]. Patients were instructed to sequentially swallow testing diets of nectar (viscosity = 295 mPas), thin liquid (21 mPas), and pudding (3682 mPas), and each testing diet had a volume sequence of 5, 10, or 20 ml, yielding a factorial design of three diet types and three volumes. If patients completed all of the swallowing protocols without oral or pharyngeal residue, piecemeal deglutition, voice changes, cough, or a decrease in oxygen saturation ≥ 3%, they passed the safe swallow pathway (no dysphagia or aspiration) test.

#### WSTs

WST was performed with a sequence of 3, 15, and 30 ml. Three trials were obtained for each WST volume, with an inter-trial-interval of 10 s. For each trial, the subjects first held water in their mouths, and then sEMG recording began. Two seconds later, the subjects were instructed to swallow the water in single swallowing act (single swallowing), if possible. They then raised their hands after they finished the swallowing tasks, and then the recording stopped. However, if a subject was unable to swallow 30 ml water in single swallowing, piecemeal deglutition was allowed.

#### Analysis

The raw EMG trace for each trial was visually inspected. Each trial was assigned as high-quality or low-quality. Low-quality trials showed noise interference in bilateral EMG trace (channels) that caused a low signal-to-noise level and was difficult to be identified symmetrical muscle activities in bilateral channels of masticatory and suprahyoid muscles (Fig. 1B). Subjects who had more than one-third their trials be of low-quality were excluded from the analysis.

The baseline interval of the sEMG signals was defined as the first 500 ms after the recording started. The mean and standard deviation (SD) of the amplitude were calculated to detect the onset and offset time of muscle activities, which was defined as when the EMG RMS trace starts to exceed and returns to the threshold level (defined as the mean + 2 SDs of amplitude during the baseline interval), respectively. The peak time was defined as the timing of the maximal amplitude of the EMG RMS trace. The time intervals were measured between the peak time of the anterior temporalis and suprahyoid activities (T-SH interval) and the masseter and suprahyoid activities (M-SH interval). The values of the peak-time intervals (T-SH or M-SH interval) were defined as the peak time of suprahyoid activities minus masticatory activities (Fig. 1C), which presented as the temporal coordination of masticatory and suprahyoid muscle contraction during swallowing. The values were averaged across the three trials. In this study, the intervals between the onset of masticatory and suprahyoid muscles were not used to represent temporal coordination performance as these parameters do not precisely respond to the actual muscle contraction [35] and are prone to be confounded by the threshold level selection.

The present study applied 30 ml WST as the stress test, which is higher than the 20 ml WST used in previous studies [25, 38]. One article [38] reported that male subjects could comfortably sip more than 30 ml of water in single swallowing. For the 30 ml WST, some subjects found it difficult to swallow in single swallowing so that we used that to categorize subjects into two subgroups: the single-swallowing (the subjects who swallowed 30 ml water with single muscle activity in each sEMG trace (channel) in at least two out of three trials) and piecemeal (all other subjects) groups (Fig. 1D). Since contraction of suprahyoid muscles dominantly contribute to swallowing acts, in piecemeal group, the suprahyoid activities with the highest maximal-amplitude were selected as the major swallowing activities whose T-SH and M-HS intervals were represent the performance of swallowing 30 ml water (Fig. 1D). Subjects with different swallowing strategies may also present different temporal coordination characteristics.

### Statistics

The demographic data was tested by Pearson's chi-squared test. The data did not follow a normal distribution so non-parametric methods were used in the analysis. All interval medians were verified by a one-sample Wilcoxon signed-rank test to analyze the skewness distribution (*H*_0_: median = 0). The correlation of age to the T-SH and M-SH intervals was analyzed using the Spearman's rank-order correlation. In a subgroup analysis, the same correlation analysis was also performed in the single-swallowing and piecemeal groups. The temporal parameters between the two groups were compared with the Mann–Whitney U test. Significance was defined as *p* < 0.05.

## Results

### The Demographic and Descriptive Information

A total of 101 subjects who met the inclusion and exclusion criteria were enrolled. Three subjects were excluded for failure to pass the safe swallow pathway of V-VST or continuous coughing during the WSTs. Another 23 subjects were excluded for having severe noise interference in more than one-third of the recording data (Fig. 2).

The age of the remaining 75 subjects (male = 26, female = 49) ranged from 65 to 88 years old (median and 25th ~ 75th percentile: 70 (67 ~ 78)) (Table 1). Most of the subjects had more than 10 pairs of occlusal teeth (median and 25th ~ 75th percentile: 14 (11 ~ 16)) with bilaterally symmetric distribution. The medians of the T-SH and M-SH intervals in all WSTs (Table 1) were higher than zero (all *p* < 0.05 in skewness verification with H_0_: median = 0), indicating that the peak time of the masticatory muscles was mostly recorded before the peak time of the suprahyoid muscles.

**Table 1:** Demographic data and the peak-time intervals between masticatory and suprahyoid activities, including the skewness verification in all subjects

	All subjects (*n* = 75) (Median, 25th ~ 75th percentile)	*P*-value
Age	70 (67 ~ 78)	
Sex (male/female)	26/49	
Pair number of occlusal teeth	14 (11 ~ 16)	
Rt side	7 (5 ~ 8)	
Lt side	7 (6 ~ 8)	
3 ml WST		
T-SH interval, Lt	0.083 (− 0.183 ~ 0.660)	< 0.01**
T-SH interval, Rt	0.090 (− 0.123 ~ 0.600)	0.012*
M-SH interval, Lt	0.250 (− 0.073 ~ 0.977)	< 0.01**
M-SH interval, Rt	0.077 (− 0.133 ~ 0.357)	0.017*
15 ml WST		
T-SH interval, Lt	0.203 (− 0.143 ~ 0.680)	< 0.01**
T-SH interval, Rt	0.143 (− 0.150 ~ 0.873)	< 0.01**
M-SH interval, Lt	0.223 (− 0.020 ~ 0.950)	< 0.01**
M-SH interval, Rt	0.107 (− 0.057 ~ 0.543)	< 0.01**
30 ml WST		
T-SH interval, Lt	0.063 (− 0.350 ~ 0.913)	0.040 *
T-SH interval, Rt	0.170 (− 0.25 ~ 1.067)	< 0.01**
M-SH interval, Lt	0.303 (− 0.097 ~ 1.650)	< 0.01**
M-SH interval, Rt	0.120 (− 0.117 ~ 0.670)	< 0.01**

*WST* water swallowing test, *Lt* left, *Rt* right, *T-SH* anterior temporalis and suprahyoid muscles, *M-SH* masseter and suprahyoid muscles

*P < 0.05, **P < 0.01 (The results of skewness verification with null hypothesis: median = 0)

### The Correlation Between Temporal Coordination and Age

Correlation analysis showed that, in 30 ml WST, the bilateral T-SH (left: *r* = 0.249, *p* = 0.031; right: *r* = 0.412, *p* < 0.01) and right M-SH (*r* = 0.242, *p* = 0.037) intervals were positively correlated with age and the degrees of the correlation were low-to-moderate *r*-value range: 0.242–0.412 (Table 2). However, the T-SH or M-SH intervals in response to the 3 or 15 ml WSTs were not significantly correlated with age. Before excluding poor quality data (*n* = 98), the right T-SH (r = 0.383, *p* < 0.01) and right M-SH (*r* = 0.233, *p* = 0.02) intervals in the 30 ml WST were positively correlated with age.

**Table 2:** The results of correlation analysis between age and the peak-time intervals of the masticatory and suprahyoid muscle activities in all subjects

	Age	
	*r*	*P* value
3 ml WST		
T-SH interval, Lt	0.188	0.106
T-SH interval, Rt	0.198	0.088
M-SH interval, Lt	0.114	0.329
M-SH interval, Rt	0.100	0.395
15 ml WST		
T-SH interval, Lt	0.168	0.150
T-SH interval, Rt	0.071	0.548
M-SH interval, Lt	0.188	0.105
M-SH interval, Rt	0.214	0.065
30 ml WST		
T-SH interval, Lt	0.249	0.031*
T-SH interval, Rt	0.412	< 0.01**
M-SH interval, Lt	0.203	0.080
M-SH interval, Rt	0.242	0.037*

*r* coefficient, *WST* water swallowing test, *Lt* left, *Rt* right, *T-SH* anterior temporalis and suprahyoid muscles, *M-SH* masseter and suprahyoid muscles

*P < 0.05, **P < 0.01

### The Comparison of Subgroups in the Demographic and Descriptive Information

**Fig. 3:**
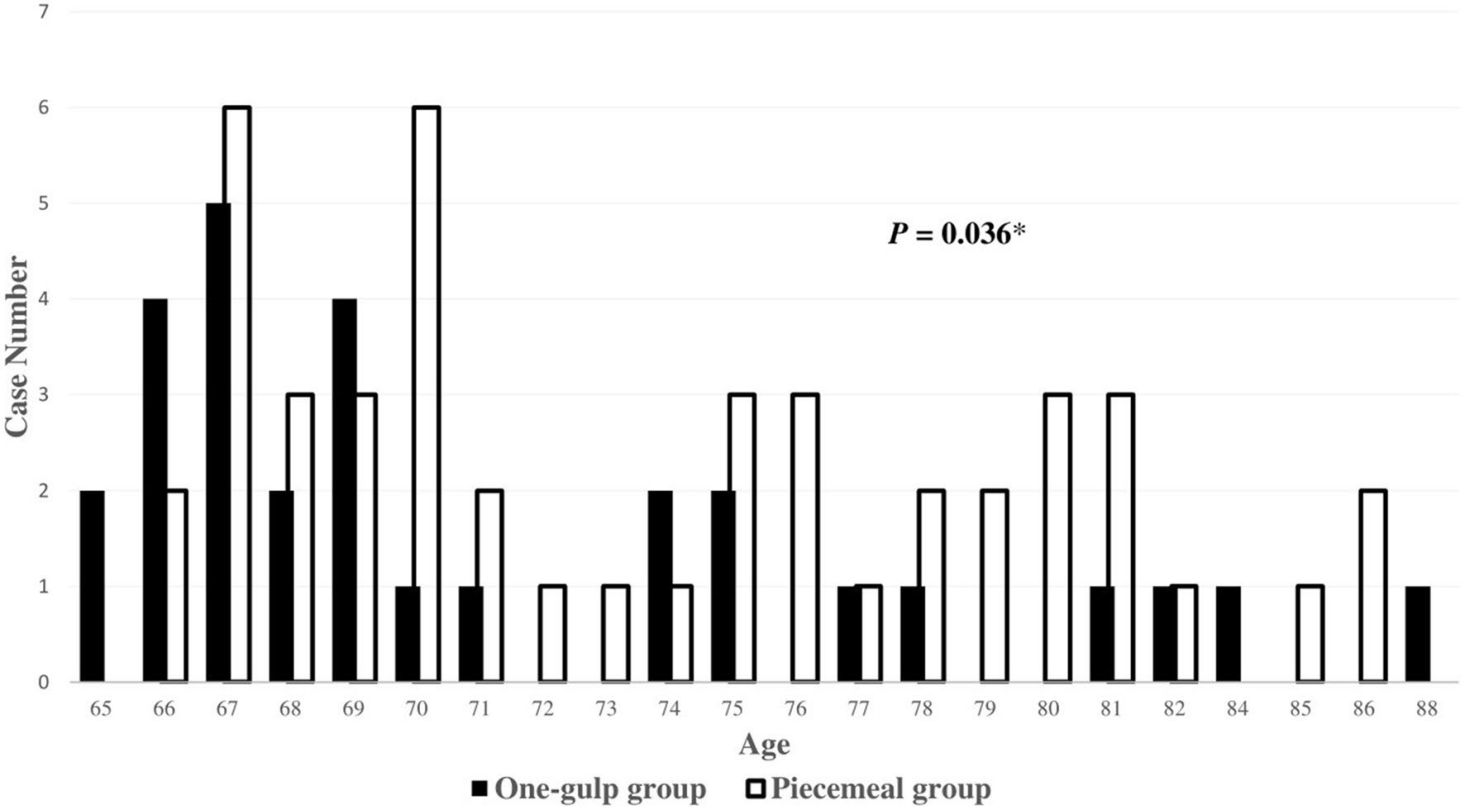
Comparison of the age distribution between the one-gulp and piecemeal groups

Subgroup analysis showed that, as compared with the single-swallowing group, the piecemeal group was significantly older (*p* = 0.036) and had a higher proportion of male patients (*p* = 0.043) (Table 3). The age distribution of the two subgroups (Fig. 3) showed that most subjects were younger than 70 years of age and the piecemeal group had a higher proportion of subjects in the old-old and oldest-old age groups (> 75 years old). The time intervals did not significantly differ between the two groups in all parameters (*p* > 0.05) in all tests, although the demographics of the two groups differed across the three WST volumes. Only the left M-SH interval in the 30 ml WST showed a tendency of significance between the two groups (*p* = 0.098), suggesting that subjects with different swallowing strategies might show different temporal coordination of the masticatory and suprahyoid muscles.

**Table 3:** Comparison of demographic data [median (25th ~ 75th percentile)] and the peak-time intervals between the one-gulp and piecemeal groups

	One-gulp group (*n* = 29)	Piecemeal group (*n* = 46)	*P* value
Age	69 (67 ~ 75)	72.5 (68.8 ~ 79)	0.036*
Sex (male/female)	6/23	20/26	0.043*
3 ml WST			
T-SH interval, Lt	0.010 (− 0.203 ~ 0.303)	0.165 (− 0.153 ~ 0.975)	0.240
T-SH interval, Rt	0.107 (− 0.080 ~ 0.433)	0.008 (− 0.201 ~ 0.650)	0.935
M-SH interval, Lt	0.140 (− 0.040 ~ 0.463)	0.375 (− 0.103 ~ 1.174)	0.265
M-SH interval, Rt	0.070 (− 0.252 ~ 0.347)	0.095 (− 0.111 ~ 0.392)	0.510
15 ml WST			
T-SH interval, Lt	0.120 (− 0.115 ~ 0.455)	0.315 (− 0.197 ~ 0.912)	0.628
T-SH interval, Rt	0.077 (− 0.128 ~ 0.820)	0.252 (− 0.198 ~ 1.188)	0.832
M-SH interval, Lt	0.217 (− 0.033 ~ 0.458)	0.300 (− 0.025 ~ 2.057)	0.217
M-SH interval, Rt	0.063 (− 0.092 ~ 0.388)	0.118 (− 0.055 ~ 0.561)	0.609
30 ml WST			
T-SH interval, Lt	0.097 (− 0.227 ~ 1.032)	− 0.002 (− 0.493 ~ 0.879)	0.609
T-SH interval, Rt	0.170 (− 0.268 ~ 0.915)	0.267 (− 0.251 ~ 1.369)	0.437
M-SH interval, Lt	0.133 (− 0.088 ~ 0.842)	0.683 (− 0.098 ~ 2.332)	0.098
M-SH interval, Rt	0.087 (− 0.115 ~ 0.273)	0.175 (− 0.219 ~ 0.899)	0.236

*WST* water swallowing test, *Lt* left, *Rt* right, *T-SH* anterior temporalis and suprahyoid muscles, *M-SH* masseter and suprahyoid muscles

*P < 0.05

### The Correlation Between Temporal Coordination and Age in Subgroup Analysis

The correlation of sex and all peak-time intervals was insignificant in both the single-swallowing and piecemeal groups (the range of R-values: -0.001 ~ 0.224, the range of p values: 0.053 ~ 0.091). In the single-swallowing group, the correlation of age and the peak-time intervals was significantly positive in the left M-SH intervals (*r* = 0.373, *p* = 0.046) responding to the 15 ml WST (Table 4). In the piecemeal group, a significant, positive correlation between peak-time intervals and age was demonstrated in right T-SH intervals (*r* = 0.477, *p* = 0.001) responding to the 30 ml WST.

**Table 4:** Correlation between age and the peak-time intervals in the one-gulp and piecemeal groups

	Group			
	One-gulp group		Piecemeal group	
	*r*	*P* value	r	*P* value
3 ml WST				
T-SH interval, Lt	0.157	0.415	0.165	0.272
T-SH interval, Rt	0.244	0.202	0.222	0.138
M-SH interval, Lt	0.051	0.795	0.064	0.673
M-SH interval, Rt	0.158	0.412	0.033	0.830
15 ml WST				
T-SH interval, Lt	0.291	0.126	0.087	0.565
T-SH interval, Rt	0.116	0.548	0.066	0.665
M-SH interval, Lt	0.373	0.046*	0.052	0.731
M-SH interval, Rt	0.156	0.419	0.207	0.166
30 ml WST				
T-SH interval, Lt	0.270	0.157	0.274	0.066
T-SH interval, Rt	0.331	0.079	0.477	0.001**
M-SH interval, Lt	0.353	0.060	0.063	0.676
M-SH interval, Rt	0.136	0.480	0.268	0.072

*r* correlation coefficient, *WST* water swallowing test, *Lt* left, *Rt* right, *T-SH* anterior temporalis and suprahyoid muscles, *M-SH* masseter and suprahyoid muscles

*P < 0.05, **P < 0.01

## Discussion

In this study, we analyzed sEMG for masticatory and larynx-elevating (suprahyoid) muscles in healthy elderly subjects performing water swallowing tasks. We observed an increase in the dispersion of the peak-time intervals with age and similar results were found in both the single-swallowing and piecemeal groups. Importantly, this increase was most pronounced in the 30 ml WST, indicating stress testing is necessary to verify the aging effect on temporal coordination of masticatory and suprahyoid activities during swallowing. The observed correlation coefficients inferred a low-to-moderate degree of correlation, suggesting that normal aging only has mild effects on changes to swallowing physiology [21]. This study is the first to address the aging effect on the temporal coordination of masticatory and larynx-elevating muscles during water swallowing. The observed dispersion increase of temporal coordination implies alternating motor control during swallowing.

Masticatory muscle electrical activity was detected slightly earlier than the suprahyoid muscle at this stage [19], which is directly controlled by the swallowing center, interacts with the masticatory center [39] and is modulated by the supratentorial level [40]. Several studies used sEMG to verify the CNS coordination of these muscle activities. Hiraoka reported the biphasic change of the masseter amplitude during swallowing and indicated the interaction between masticatory and swallowing centers [22]. Takeda and Saitoh showed the correlation of the masseter activities of clenching before swallowing and the time interval between the onset of suprahyoid activities and the onset of the thyroid cartilage’s anterior shift, and urged that subcortex coordinated the sequence of oral and pharyngeal phases [23]. Yoneda and Saitoh revealed the crucial role of the time interval between the peak of masticatory and suprahyoid activities during mastication on switching from the oral phase to the pharyngeal phase and suggested the switching process controlled by the “convertor neurons” [24]. Those studies supported that the muscle activities of masticatory and suprahyoid co-contraction during swallowing may indicate the CNS coordination. Moreover, imaging demonstrated the aging cortex’s alternative activities during swallowing [41–43], which may elongate the CNS’s control circuits and further accounts for the increasing temporal dispersion of masticatory and suprahyoid muscle co-contraction. sEMG has been used as a tool to screen for adequate swallowing function and dysphagia, but was applied only on a limited basis to investigate the effects of normal aging on swallowing function. Vaiman et al. recorded the activities of the orbicularis oris, masseter, suprahyoid, and infrahyoid muscles. The mean and range amplitude of those muscles were measured and the duration of the swallowing reflex was determined as the time intervals between the onset of orbicularis oris activity and the offset of masseter activity. This revealed that the range amplitude of the suprahyoid muscles rather than the swallowing reflex significantly decreased with age [25], but the swallowing reflex duration was significantly longer in the older group (those > 70 years old) compared to their younger counterparts (< 70 years old) [21]. Chen and Lin recorded the activities of the orbicularis oris inferior, suprahyoid, and infrahyoid muscles in community-dwelling older adults (over 65 years of age) and determined the starting time of the swallowing reflex as the interval between the onset of orbicularis oris inferior and suprahyoid muscles, and the total swallowing time as the interval of onset of suprahyoid and offset of infrahyoid. This reported that the total swallowing time rather than the starting time of the swallowing reflex was significantly shorter in the 60 ~ 69-year-old age group compared with the 70 ~ 79 and 80 ~ 89-year-old age groups [31]. Ding et al. also recorded the activities of the orbicularis oris inferior, suprahyoid, and infrahyoid muscles and determined the start time of suprahyoid and infrahyoid as the interval between the onset of orbicularis oris inferior and suprahyoid or infrahyoid muscles, respectively. This demonstrated that the start time of the suprahyoid was significantly longer in the older group compared with the young group [32]. The study designs of the work by Chen & Lin and Ding et al. were similar to ours, and the results of Ding et al. support our findings. Both studies chose temporal intervals between the orbicularis oris and suprahyoid or infrahyoid muscles rather than masticatory and suprahyoid muscles. They also chose the onset time of muscle activities rather than peak time, which may be driven by different neurophysiological mechanisms. This may account for their inconsistent results. The co-work of the masticatory and suprahyoid muscles during masticatory and swallowing movement phases has been proven in function [22], neuroanatomy [40], and experimental research [22–24, 26], so the temporal intervals of masticatory and suprahyoid activities could more appropriately address coordination performance.

Our study revealed a volume effect, even in the subgroup analysis, and similar results have been reported in previous studies [25, 44–46]. Increasing bolus volume induced a stronger amplitude and a longer duration of muscle activities. In our experiment procedure, we increased the volume of water and may have “overstressed” the swallowing function of participants with the 30 ml dose, while swallowing 20 ml of water in single swallowing was thought of as a stress test [25, 38]. Similar results in the piecemeal group indicate that elderly individuals might feel stress in the 30 ml WST because they had to try their best to swallow the water in single swallowing. This may be accounted for the conscious control needed during the initial stage of swallowing [40]. Therefore, we assumed that the volume stress was crucial for testing how aging changes swallowing performance in healthy elderly individuals. The optimal volume warrants further study.

Combining sEMG and our study design, we observe minor changes in swallowing physiology with age in healthy elderly individuals. However, our study had several limitations. First, we used V-VST, not VFSS, to determine dysphagia. Although the V-VST has a good sensitivity (88.2%) and specificity (64.7%) for detecting clinical signs of aspiration or penetration, mild dysphagic elders might not be excluded. This affected the interpretation of the results. In our study design, we partially minimized this limitation by excluding subjects who coughed during any step of the WSTs. Second, group comparison between young and old age population was not conducted and the age distribution was unequal. Since aging influences changes in swallowing physiology, the unequal age distribution certainly affected the results. However, there were fewer cases over 70 years of age than younger than 70 years of age. We inferred that increasing the number of older individuals would enhance the significance of the aging effect. Third, our study had an unbalanced sex proportion, but the correlation of sex and the peak-time intervals was insignificant, analogous to the results of previous studies [21, 31, 47]. Fourth, other factors, such as asymmetric activities during contraction of masticatory muscles [48], may affect the measurement of temporal coordination between masticatory and suprahyoid muscles. The confounding effect of other factors should be verified in further study. Fifth, we subjectively, not objectively, screened the data quality by noise interference for excluding poor quality data. The noise interference partially resulted from the disadvantages of sEMG recording, such as variant skin impedance, the variant distance from electrodes to target muscles, and the incident locating deviation from the muscle belly, especially in subjects with loose skin and subcutaneous tissue. However, before excluding poor quality data (*n* = 98), the peak-time intervals of right T-SH and right M-SH in the 30 ml WST still had a significantly positive correlation with age. The data extraction could amplify the significance of the aging effect on the temporal coordination between masticatory and suprahyoid muscles.

## Conclusion

sEMG can be applied to investigate the effect of aging on swallowing function. The temporal dispersion of the T-SH and M-SH intervals representing coordination performance was positively correlated with age, suggesting these parameters can be used to monitor the functional changes of swallowing in the elderly. They may reflect CNS control, especially in elderly individuals with subclinical neurodegenerative disorders. Further study is needed to verify the normative values of these parameters in different age groups.
